# Construction and analysis of a ceRNA network and patterns of immune infiltration in chronic rhinosinusitis with nasal polyps: based on data mining and experimental verification

**DOI:** 10.1038/s41598-022-13818-6

**Published:** 2022-06-13

**Authors:** Jing-cai Chen, Qi-long Xing, Hui-wen Yang, Fan Yang, Yao Luo, Wei-jia Kong, Yan-jun Wang

**Affiliations:** 1 Department of Otorhinolaryngology, Union Hospital, Tongji Medical College, Huazhong University of Science and Technology, Wuhan, China; 2 Institute of Otorhinolaryngology, Union Hospital, Tongji Medical College, Huazhong University of Science and Technology, Wuhan, China; 3 School of Computer Science and Technology, Huazhong University of Science and Technology, Wuhan, China; 4 Department of Otorhinolaryngology, The First Affiliated Hospital, School of Medicine, Shihezi University, Shihezi, Xinjiang China

**Keywords:** Gene regulatory networks, Chronic inflammation, Diagnostic markers, Chronic inflammation

## Abstract

Recent studies have revealed the significant role of the competing endogenous RNA (ceRNA) network in human diseases. However, systematic analysis of the ceRNA mechanism in chronic rhinosinusitis with nasal polyps (CRSwNP) is limited. In this study, we constructed a competitive endogenous RNA (ceRNA) network and identified a potential regulatory axis in CRSwNP based on bioinformatics analysis and experimental verification. We obtained lncRNA, miRNA, and mRNA expression profiles from the Gene Expression Omnibus. After analysis of CRSwNP patients and the control groups, we identified 565 DE-lncRNAs, 23 DE-miRNAs, and 1799 DE-mRNAs by the DESeq2 R package or limma R package. Enrichment analysis of 1799 DE-mRNAs showed that CRSwNP was associated with inflammation and immunity. Moreover, we identified 21 lncRNAs, 8 miRNAs and 8 mRNAs to construct the lncRNA-miRNA-mRNA ceRNA network. A potential MIAT/miR-125a/IRF4 axis was determined according to the degree and positive correlation between a lncRNA and its competitive endogenous mRNAs. The GSEA results suggested that IRF4 may be involved in immune cell infiltration. The validation of another dataset confirmed that MIAT and IRF4 were differentially expressed between the CRSwNP and control groups. The area under the ROC curve (AUC) of MIAT and IRF4 was 0.944. The CIBERSORT analysis revealed that eosinophils and M2 macrophages may be involved in the CRSwNP process. MIAT was correlated with dendritic cells and M2 macrophages, and IRF4 was correlated with dendritic cells. Finally, to validate the key genes, we performed in-silico validation using another dataset and experimental validation using immunohistochemistry, immunofluorescence, and Western blot. In summary, the constructed novel MIAT/miR-125a/IRF4 axis may play a critical role in the development and progression of CRSwNP. We believe that the ceRNA network and immune cell infiltration could offer further insight into novel molecular therapeutic targets for CRSwNP.

## Introduction

Chronic rhinosinusitis (CRS) is a common chronic disease with a heavy socioeconomic burden^1^. CRS is a multifactorial and heterogeneous group of sinus diseases characterized by inflammatory disease of the sinuses and mucosa of the nose^2^. CRS is divided into CRS with nasal polyps (CRSwNP) and CRS without nasal polyps (CRSsNP) based on the presence or absence of polyps^3^. Clinically, CRSwNP is more recalcitrant and refractory than CRSsNP^4^. CRSwNP is histologically characterized by stromal tissue edema, formation of pseudocysts, and significant immune cell infiltration^5^. CRSwNP is classically divided into two subsets: eosinophilic nasal polyps (ECRSwNP), which are found mainly in European and American patients and feature a Th2 signature, and noneosinophilic nasal polyps (non-ECRSwNP), which are found mainly in Asian patients with a mixed Th1/Th17 pattern of inflammatory response^6,7^. Despite the great advances in functional endoscopic sinus surgery (FESS)^8^ and biologics^9,10^, the treatment effect of CRSwNP is still unsatisfactory. There remained a surgical revision rate of 21%–29.9% for CRSwNP^11,12^. While biologic therapy has been deployed with encouraging results, its use is constrained by high costs and the risk of anaphylaxis^13,14^. Recently, accumulating studies have demonstrated that sinonasal epithelial cell barrier defects, increased exposure to pathogenic and colonized bacteria, and dysregulation of the host immune system are all thought to play prominent roles in disease pathogenesis^15,16^. Although insights into the pathophysiology of CRSwNP have largely expanded over the last few decades, the precise etiology and mechanism of persistence are still unknown. Therefore, a more comprehensive understanding of the etiopathogenesis of CRSwNP helps to provide novel potential targets and clinical strategies for CRSwNP treatment.

Due to the development of sequencing and bioinformatics approaches, the important functions of noncoding RNAs (ncRNAs) have been identified in various aspects of cell biology and life processes. NcRNAs include microRNAs (miRNAs), long noncoding RNAs (lncRNAs), pseudogenes and circular RNAs, which were previously called “dark matter” or “junk RNA” because of their inability to encode proteins^17^. The study of dysregulated ncRNAs in diseases may provide a better understanding of the pathogenesis and hold promise for therapeutic strategies^18^. MiRNAs are small noncoding RNAs (21–23 nucleotides) with a distinct capacity to negatively regulate target gene expression at the posttranscriptional level^19^. Increasing evidence indicates that miRNAs play a critical role in the pathogenesis of CRSwNP. A previous study revealed that the elevated level of miR-125b contributes to mucosal eosinophilia recruitment via the enhancement of type I interferon (IFN) expression in ECRSwNP tissue and may play a critical role in airway antiviral innate immunity by suppressing EIF4E-binding protein 1 (4E-BP1) expression^20^. Furthermore, miR-124 participates in the regulation of the inflammatory response by mediating the expression of aryl hydrocarbon receptor (AHR) in CRSwNP^21^. Moreover, let-7a-5p has been revealed to regulate the inflammatory response by interacting with IL-6 through the Ras-MAPK pathway in CRSwNP^22^.

LncRNAs are a class of noncoding transcripts of > 200 nucleotides that can regulate gene expression at the transcriptional or posttranscriptional level^23^. The hypothesis of competing endogenous RNAs (ceRNAs) states that lncRNAs and mRNAs can interact with each other via miRNAs by forming a regulatory network^24^. According to this theory, lncRNAs act as miRNA sponges by miRNA response elements (MREs) to absorb and bind miRNAs, which can modulate mRNA expression^24,25^. The expression levels of these two RNA transcripts will have a positive relationship with each other^26^. The effects of ceRNA networks depend on the identity, concentration, and subcellular distribution of the RNA and the miRNA species. However, lower levels of ncRNAs may also contribute to ceRNA sponge mechanisms, and even ceRNAs undergo marked rewiring between normal and pathological tissues^27,28^. Many previous studies have confirmed that lncRNAs play essential roles in the proliferation, metastasis, drug sensitivity, and progression of tumors^29,30^. Over the last few years, many studies have validated the ceRNA theory, as emerging evidence has verified that ceRNA crosstalk imbalance is associated with various diseases, especially cancers^26–28^. Recent research has expanded to establish ceRNA networks in the field of inflammatory diseases^31^. Regarding chronic nasal inflammation, limited data about the role of lncRNAs have been reported, and only a limited number of lncRNAs have been discovered to be involved in its pathogenesis. Wang et al. found that lncRNA XLOC_010280 was highly expressed in ECRSwNP and played a vital role in eosinophilic inflammation by regulating CCL18 expression^32^. Another study indicated that lncRNA NEAT1 and its targets (miR-21 and miR-125a) were strongly correlated with the disease risk, severity, and inflammation of allergic rhinitis^33^. However, to understand the roles of ceRNA networks in the pathogenesis and pathological conditions of CRSwNP, further research is needed.

In this study, we hypothesized that there were still a significant number of previously unexplored genetic differences between CRSwNP and normal tissues, especially with respect to ncRNA expression patterns. We screened several differentially expressed mRNAs, miRNAs and lncRNAs between CRSwNP patients and healthy controls from the Gene Expression Omnibus (GEO) dataset. Then, functional enrichment analysis was conducted to assess the functional role and potential mechanism of differentially expressed mRNAs in CRSwNP. Moreover, a ceRNA network was constructed to elucidate the interactions and potential crosstalk among the key lncRNAs, miRNAs, and mRNAs in CRSwNP. Finally, immune infiltration analysis was performed to study the relationship between the ceRNA axis (lncRNA and mRNA) and infiltrating immune cells to better understand the molecular immune mechanism during the development of CRSwNP. Subsequent to in-silico analysis, experimental validation of the key mRNAs was performed. Our study aimed to comprehensively assess the correlation of ceRNAs with the immune microenvironment, diagnostic biomarkers, and therapeutic targets in CRSwNP patients.

## Materials and methods

### Datasets selection and raw data preprocessing

**Figure 1:**
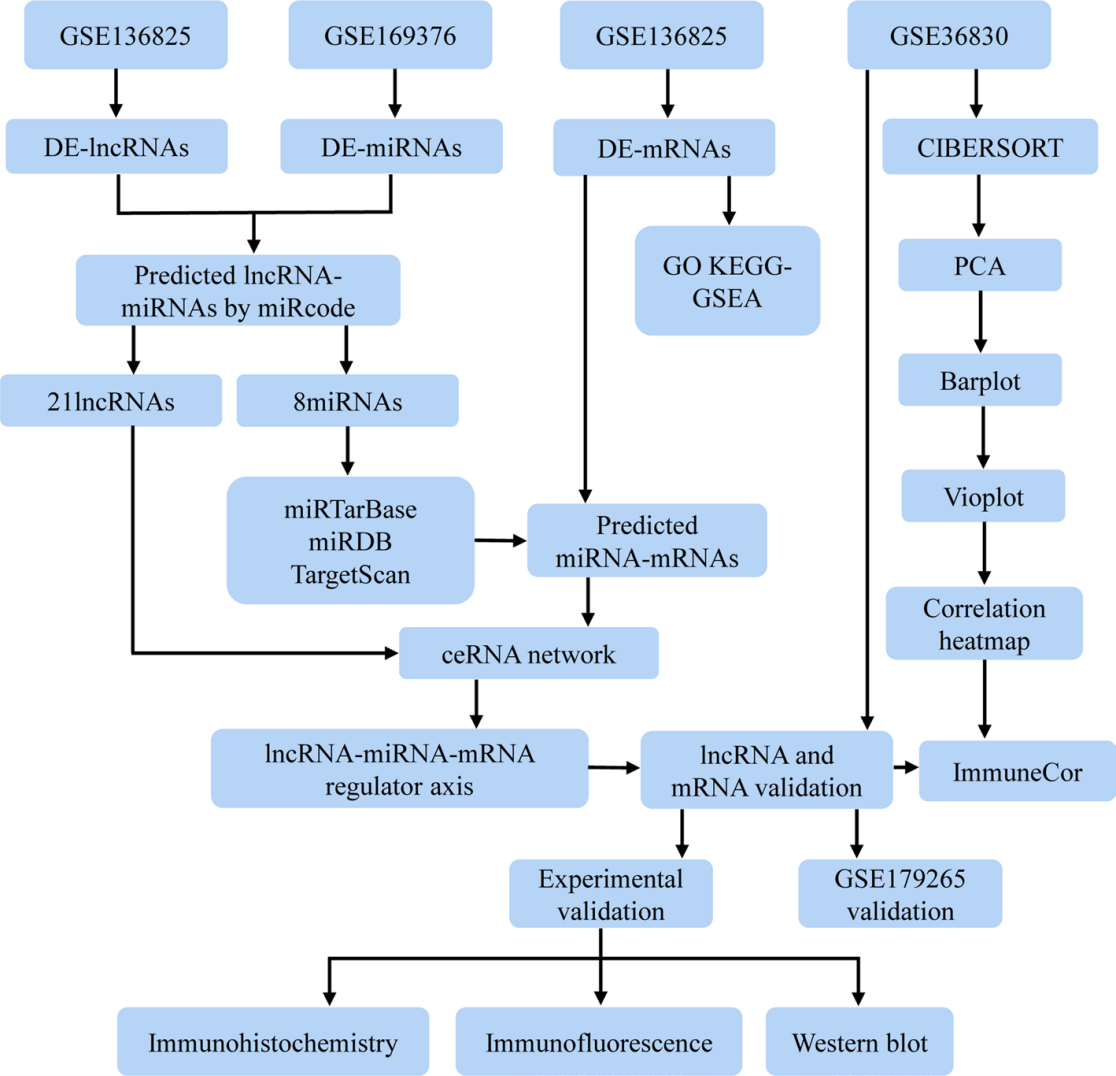
Flow chart of the study design.

The study was carried out in compliance with the guidelines of the Declaration of Helsinki. Figure 1 shows the workflow chart of this study. The Bioconductor package “GEOquery”^34^ of R software (version 4.1.0, http://r-project.org/) was used to extract the CRSwNP expression profile datasets GSE136825^35^, GSE36830^36^, GSE179265^37^ and GSE169376^38^ from the Gene Expression Omnibus (GEO) database (https://www.ncbi.nlm.nih.gov/geo/). The mRNA and lncRNA data from GSE136825 and the miRNA data from GSE169376 were sorted into standardized original data for subsequent analysis. The GSE36830 dataset and GSE179265 dataset were applied to examine the expression of lncRNA and mRNA biomarkers as the validation cohort. The GSE36830 dataset was applied to analyze immune cell infiltration. The ensemble ID of samples was converted by using the HGNC symbol. Detailed information on the four datasets is shown in Table 1.

**Table 1:** Details for GEO CRSwNP Data.

Accession	Platform	Study type	Sample	CRSwNP/Control	RNA type
GSE136825	GPL20301	RNA-seq	NP/Control	42/28	mRNA/lncRNA
GSE36830	GPL570	Array	NP/Control	6/6	mRNA/lncRNA
GSE179265	GPL24676	RNA-seq	NP/Control	17/7	mRNA/lncRNA
GSE169376	GPL21572	Array	NP/Control	6/3	microRNA

*CRSwNP* chronic rhinosinusitis with nasal polyps; *NP* nasal polyp; *GEO* Gene Expression Omnibus.

### Identification of differentially expressed genes (DEGs)

We used the “DESeq2” package^39^ to identify DEGs (DE-lncRNAs and DE-mRNAs) between nasal polyp samples and control samples. We considered that |log2 FC| (absolute value of log2 in the fold change of gene expression) ≥ 1 and adjusted p value with a false discovery rate (FDR) < 0.05 were statistically significant. The “limma” package^40^ was used to identify DE-miRNAs between nasal polyp samples and control samples, and FDR < 0.05 and |log2 FC|≥ 1 were defined as the criteria of significance. Then, heat maps and volcano plots were drawn using “pheatmap” and “ggplot2” packages of R software to indicate the DEGs.

### Functional enrichment analysis

We used the R package “clusterprofiler”^41^ to analyze the Gene Ontology (GO) functional enrichment of DE-mRNAs. By functional enrichment analysis, we determined which biological process (BP), cellular component (CC) and molecular function (MF) were significantly (FDR < 0.05) enriched in both CRSwNP and control subjects during differentiation. The Kyoto Encyclopedia of Genes and Genomes (KEGG)-Gene Set Enrichment Analysis (GSEA) was searched for pathways at the significance level set at FDR < 0.05^42,43^.

### Construction of a ceRNA network

First, lncRNA-miRNA interactions were constructed based on DE-lncRNAs from miRcode (http://www.mircode.org/). The candidate target miRNAs were then compared with DE-miRNAs. Next, we used miRTarBase (https://mirtarbase.cuhk.edu.cn/~miRTarBase/miRTarBase_2022/php/index.php), miRDB (http://www.mirdb.org/) and TargetScan (http://www.targetscan.org/) to predict miRNA-mRNA interactions by candidate target miRNAs. Then, we constructed a lncRNA-miRNA-mRNA ceRNA network and visualized it by Cytoscape software (version 3.8.2, https://cytoscape.org/^44^. The Cytoscape plug-in cytoHubba was used to identify hub genes in this network. Correlations between the expression of the hub lncRNA and its competitive endogenous mRNAs were analyzed by Pearson correlation analyses.

### Gene set enrichment analysis of the key mRNAs in the ceRNA network

Gene set enrichment analysis (GSEA) is a knowledge-based method for the translation of genome-wide expression profiles^43^. We analyzed the pathways of the key mRNAs of the ceRNA network using GSEA. The cutoff criteria were NES > 2, FDR < 0.01, and *p* value < 0.01.

### Validation of the ceRNA network

To verify the ceRNA network and our results, we used the “limma” package to identify DEGs of GSE36830, which included lncRNA and mRNA information from 6 CRSwNP patients and 6 control subjects. DEGs were screened by |log2 FC|≥ 1 and FDR < 0.05. Receiver operating characteristic (ROC) curves and the area under the curve (AUC) were used to measure the diagnostic performance of the lncRNAs, mRNAs, lncRNAs and mRNAs by GraphPad Prism Version 9.

### Evaluation of immune cell infiltration

CIBERSORT is a deconvolution algorithm that uses gene expression signatures consisting of 547 genes to predict 22 immune cell phenotypes^45^. We uploaded standard annotated gene expression data of GSE36830 to the CIBERSORT web portal (http://cibersort.stanford.edu/) and obtained the immune cell infiltration matrix. The samples were screened according to *p* value < 0.05. Then, we used the “ggplot2” package^46^ to draw two-dimensional PCA maps and violin diagrams to visualize the differences in immune cell infiltration. The “corrplot” package^47^ was used to draw a correlation heatmap to visualize the correlation of 22 types of infiltrating immune cells.

### Correlation analysis between the ceRNA axis (lncRNA and mRNA) and infiltrating immune cells

Spearman correlation analysis was performed on lncRNAs, mRNAs of the ceRNA axis and infiltrating immune cells using the "ggstatsplot" package based on the lack of a linear relationship between genes and immune cells, and the gene distribution did not conform to a normal distribution. The "ggplot2" package was used to visualize the results.

### In-silico validation

To validate the mRNA and lncRNA expression levels of the screened key genes between CRSwNP and control subjects, the validation dataset GSE179265 was used. The dataset included RNA-seq expression data from human nasal polyp samples (n = 17) and control samples (n = 7). The DEGs were assessed using the default settings of “EdgeR” package^48^. DEGs were screened by |log2 FC|≥ 1 and FDR < 0.05. The expression of mRNA and lncRNA between CRSwNP and control subjects was analyzed by Student's t-test, using GraphPad Prism version 9. The correlation between the mRNA and lncRNA was analyzed by Pearson correlation analyses using GraphPad Prism Version 9.

### Experimental validation

Twenty four CRSwNP nasal polyps and twelve normal nasal mucosa tissue samples were obtained from the Department of Otorhinolaryngology, Union Hospital (Wuhan, China) and all subjects signed consent forms. Diagnosis of CRSwNP was made according to the current European Position Paper on Rhinosinusitis and Nasal Polyps 2020^1^. All control patients were defined as those without CRS who were undergoing skull base surgery or endonasal surgery for anatomic abnormalities. This study was approved by Ethics Committee of Union Hospital, Tongji Medical College, Huazhong University of Science and Technology (20,220,343).

### Immunohistochemistry and immunofluorescence

We fixed the nasal polyp tissues and control tissues in 4% paraformaldehyde and embedded it in a wax block. 5 um sections were obtained from blocks, and the sections were dewaxed with xylene and hydrated in a graded ethanol series. The samples were then treated with 3% hydrogen peroxide solution for 10 min and further washed with PBS. Antigen retrieval was performed in TRIS–EDTA buffer (pH 9.0) using a microwave oven (900 W for 15 min). The blocked sections were incubated overnight at 4 °C with anti-IRF4 (1:300 diluted, rabbit polyclonal, YT2399, ImmunoWay Biotechnology, Plano, TX, USA) and CD68 (1:4000 diluted, Mouse Monoclonal, 66231-2-Ig, Proteintech) antibody. The next day, the sections were rinsed three times with PBS, and for immunohistochemistry, the slides were incubated with horseradish peroxidase (HRP)-linked secondary antibodies of two-step immunohistochemical detection kit (G1210-2, Servicebio, Wuhan, China), and the color was visualized with DAB (Servicebio, Wuhan, China). The percentage of positive epithelial cells was categorized into four grades: 0 for < 5% positive cells, 1 for 6%–25% positive cells, 2 for 26%–50% positive cells, 3 for 51%–75% positive cells, and 4 for > 75% positive cells. Staining intensity of cytoplasm and nuclei was categorized into three grades: 0, no staining; 1, slightly yellow (weak) staining; 2, brown-yellow (moderate) staining; and 3, brown (strong) staining. The final score was the multiplication of the percentage and intensity scores (overall score range, 0–12). The positive inflammatory cells were presented as numbers per high-power filed (HPF).

For immunofluorescence histochemistry, the sections were incubated with Alexa Fluor 488 Donkey Anti-Rabbit IgG (H+L) (diluted 1:300) and Alexa Fluor 647 Donkey Anti- Mouse IgG (H+L) (diluted 1:300) for 1 h. After four washes, the slides were incubated with DAPI (4,6-diamidino-2-phenylindole, G1012-100ML, Servicebio, Wuhan, China) for the staining of nuclei. Images were captured with a confocal laser scanning microscope (Nikon-A1-Si, Nikon Corporation).

### Western blotting

Total proteins were extracted from CRSwNP tissues and control tissues using RIPA lysis buffer (Beyotime Biotechnology, China) containing phosphatase inhibitors and PMSF, followed by concentration detection with the BCA protein assay kit. 20 μg protein were separated by electrophoresis using 10% sodium dodecyl sulfate polyacrylamide gels and then transferred to polyvinylidene difluoride (PVDF) membranes for incubation with anti-IRF4 (1:1000 diluted, rabbit polyclonal, YT2399, ImmunoWay Biotechnology, Plano, TX, USA) antibody. After three washes with TBST, membranes were incubated with horseradish peroxidase-conjugated goat anti-rabbit IgG (diluted 1:5000) for 1 h at room temperature, then washed with TBST buffer three times. An ECL detection reagent (G2014-50ML, Servicebio, Wuhan, China) was used to visualize the membranes. The relative expression level of proteins was represented in the ratio of the target protein and GAPDH using ImageJ software (National Institutes of Health, USA).

### Statistical analysis

All data were expressed as the mean ± SEM. Analyses were performed using the Student’s t test and the Mann–Whitney test as appropriate, using GraphPad Prism Version 9. Differences were considered significant at *p* value < 0.05.

## Results

### Differentially expressed RNAs (DE-RNAs) in CRSwNP

**Figure 2:**
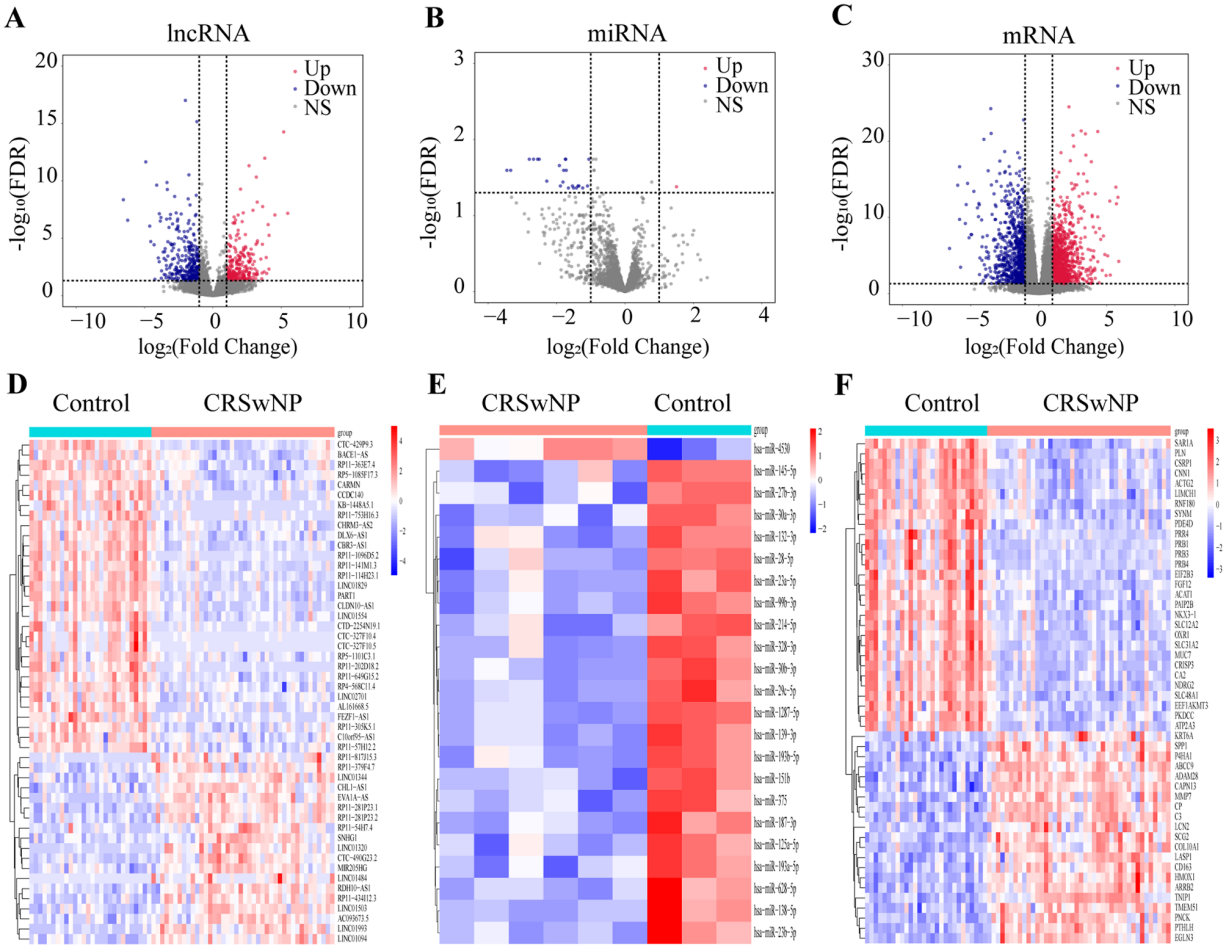
Differentially expressed gene analysis. Red indicates upregulation, blue indicates downregulation, and black indicates no significant difference. (**A**) Volcano map of significantly differentially expressed lncRNAs. (**B**) Volcano map of significantly differentially expressed miRNAs. (**C**) Volcano map of significantly differentially expressed mRNAs. (**D**) Hierarchical clustering heatmap of the top 50 differentially expressed lncRNAs. (**E**) Hierarchical clustering heatmap of all differentially expressed miRNAs. (**F**) Hierarchical clustering heatmap of the top 50 differentially expressed mRNAs. Hierarchical clustering heatmap were generated using the R package “pheatmap” (version 1.0.12. https://CRAN.R-project.org/package=pheatmap).

Using a cutoff threshold of |log2 FC|≥ 1 and FDR < 0.05 for the 42 CRSwNP tissues compared with 28 control samples, we identified 565 DE-lncRNAs (267 upregulated and 298 downregulated), 23 DE-miRNAs (1 upregulated and 22 downregulated), and 1799 DE-mRNAs (1071 upregulated and 728 downregulated). All DEGs were highlighted in volcano plots (Fig. 2A–C). Hierarchical clustering analysis revealed the expression patterns of the top 50 DE-mRNAs and DE-lncRNAs, and all DE-miRNAs were capable of distinguishing CRSwNP subjects from control subjects (Fig. 2D–F).

### Functional enrichment analysis

**Figure 3:**
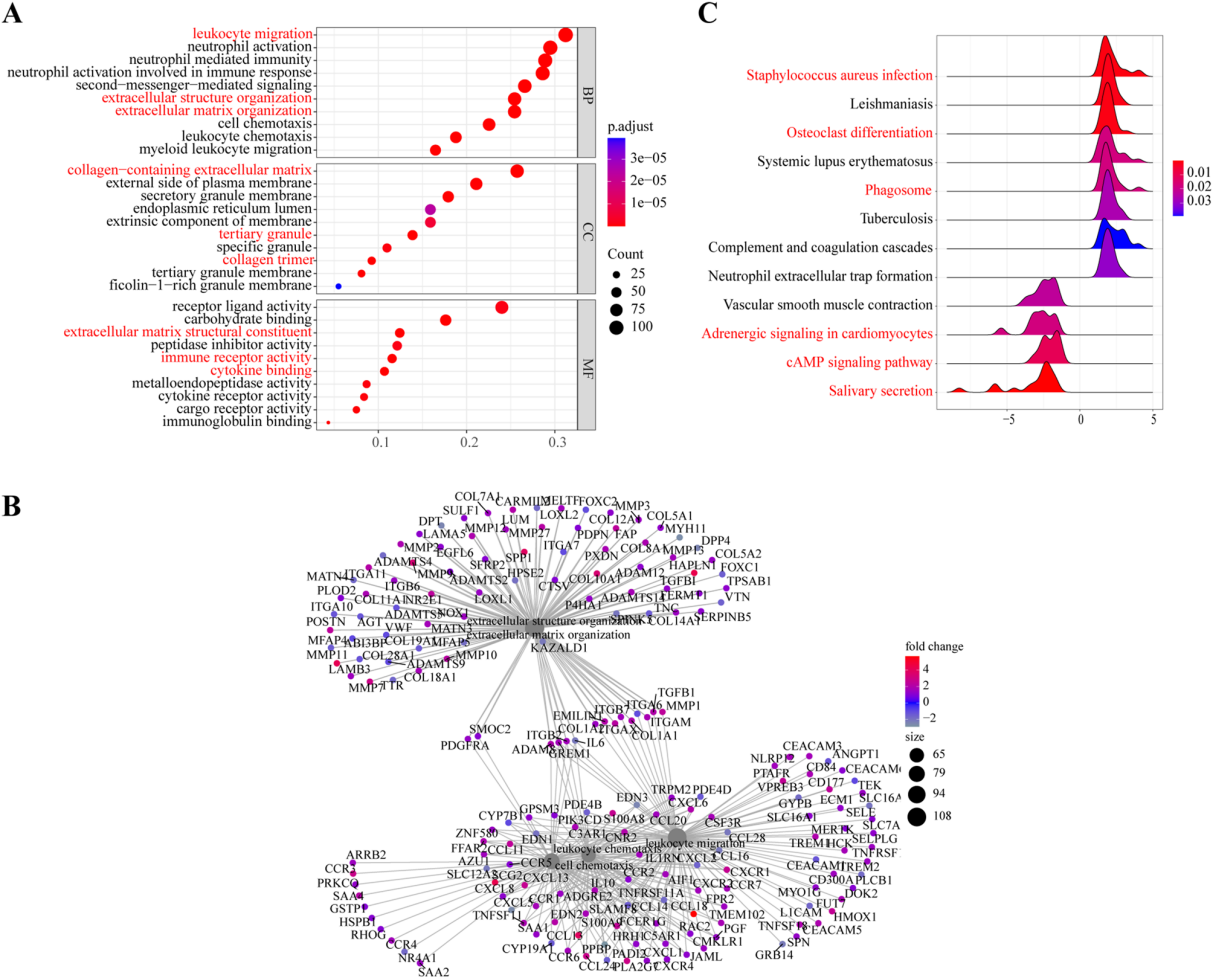
Functional enrichment analysis of DE-mRNAs. (**A**) GO analysis of DE-mRNAs in biological process (BP), molecular functions (MF) and cellular component (CC). (**B**) Gene symbols and interactions of the significantly DE-mRNAs in biological process (BP) are shown. (**C**) KEGG-GSEA pathway analysis of DE-mRNAs. GO, Gene Ontology; KEGG, Kyoto Encyclopedia of Genes and Genomes (www.kegg.jp/kegg/kegg1.html); GSEA, Gene Set Enrichment Analysis.

Gene Ontology (GO) analysis was applied to test the biological function of the identified genes. A total of 972 GO terms were enriched, and the top 10 enriched GO terms of each group according to FDR are displayed. DE-mRNAs were enriched in leukocyte migration, extracellular structure organization and extracellular matrix organization in BP, collagen-containing extracellular matrix, tertiary granule and collagen trimer in CC, and immune receptor activity, cytokine binding and extracellular matrix structural constituent in MF (Fig. 3A). Figure 3B shows the gene symbols and their interactions in BP of DE-mRNAs. The network presented numerous genes, such as CCL18, CCL13, MMP11, SCG2, HAPLN1, and SPP1, that were significantly differentially expressed in CRSwNP. Moreover, Staphylococcus aureus infection, osteoclast differentiation, and phagosome were upregulated, while the cAMP signaling pathway, salivary secretion and adrenergic signaling in cardiomyocytes were downregulated by KEGG-GSEA (Fig. 3C).

### Construction of a ceRNA network and validation of lncRNAs and mRNAs

**Figure 4:**
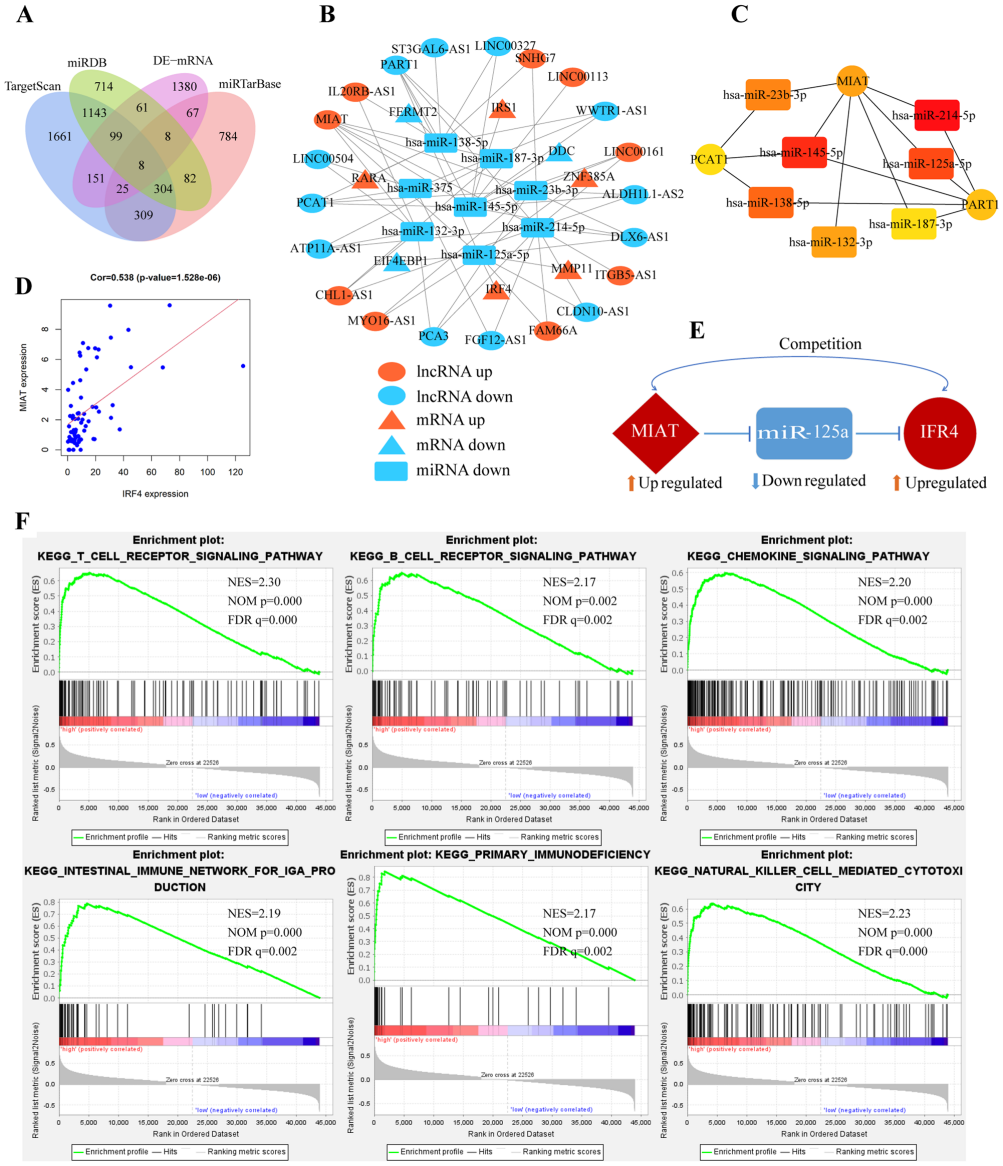
A potential lncRNA-miRNA-mRNA ceRNA network was constructed. (**A**) Venn diagram of predicted target mRNAs and DE-mRNAs. (**B**) The ceRNA network derived from DEGs in CRSwNP. The ellipses, round rectangles and triangles represent lncRNAs, miRNAs and mRNAs, respectively. Upregulation is represented by red nodes, while downregulation is represented by blue nodes. (**C**) Ten hub genes in this network using the Cytoscape plug-in cytoHubba. (**D**) Correlation analysis between MIAT and IRF4 in CRSwNP. (**E**) The MIAT/miR-125a/IRF4 regulatory axis perfectly conformed to the ceRNA hypothesis. (**F**) Gene set enrichment analysis of IRF4. DE-mRNA, differentially expressed mRNA; NES, normalized NS; FDR, false discovery rate.

First, we employed miRcode to predict lncRNA-miRNA interactions based on DE-lncRNAs. The overlapping target miRNAs between candidate target miRNAs and DE-miRNAs were chosen. We obtained 8 miRNAs and 21 lncRNAs (56 lncRNA-miRNA pairs). Next, miRTarBase, miRDB and TargetScan were utilized to predict miRNA-mRNA interactions. Then, we chose the overlapping target mRNAs (8 mRNAs) by analyzing the predicted target mRNAs and DE-mRNAs (Fig. 4A). Finally, a lncRNA-miRNA-mRNA ceRNA network was constructed with 21 lncRNAs, 8 miRNAs and 8 mRNAs, as shown in Fig. 4B. The Cytoscape plug-in cytoHubba was utilized to determine the hub regulatory network (Fig. 4C) (Table 2). The results showed that only lncRNA MIAT was chosen according to degree > 5 as the hub lncRNA. According to the ceRNA theory, the expression of lncRNAs and mRNAs are coexpression relationships. Pearson correlation coefficients were used to identify mRNA‑lncRNA pairs based on the expression values of competing mRNA-lncRNA pairs. IRF4 expression was moderately positively correlated with MIAT expression (r = 0.538, *p* value < 0.05) (Fig. 4D), while other mRNAs were not correlated with MIAT. Therefore, we consider the MIAT/miR-125a/IRF4 subnetwork to be a potential key regulatory axis in the ceRNA network, which may play a critical role in the pathogenesis of nasal polyps (Fig. 4E).

**Table 2:** The result of hub genes using Cytoscape plug-in Cytohubba.

Name	Regulation	Degree
hsa-miR-214-5p	Down	13
hsa-miR-145-5p	Down	11
hsa-miR-125a-5p	Down	10
hsa-miR-138-5p	Down	9
hsa-miR-23b-3p	Down	7
MIAT	Up	6
hsa-miR-132-3p	Down	6
PART1	Down	5
PCAT1	Down	4
hsa-miR-187-3p	Down	4

We performed GSEA to identify the pathways associated with IRF4. The expression of IRF4 was used as the basis of sample classification. GSEA showed that the T-cell receptor signaling pathway, B-cell receptor signaling pathway, chemokine signaling pathway, and natural killer cell-mediated cytotoxicity were significantly positively related (Fig. 4F).

**Figure 5:**
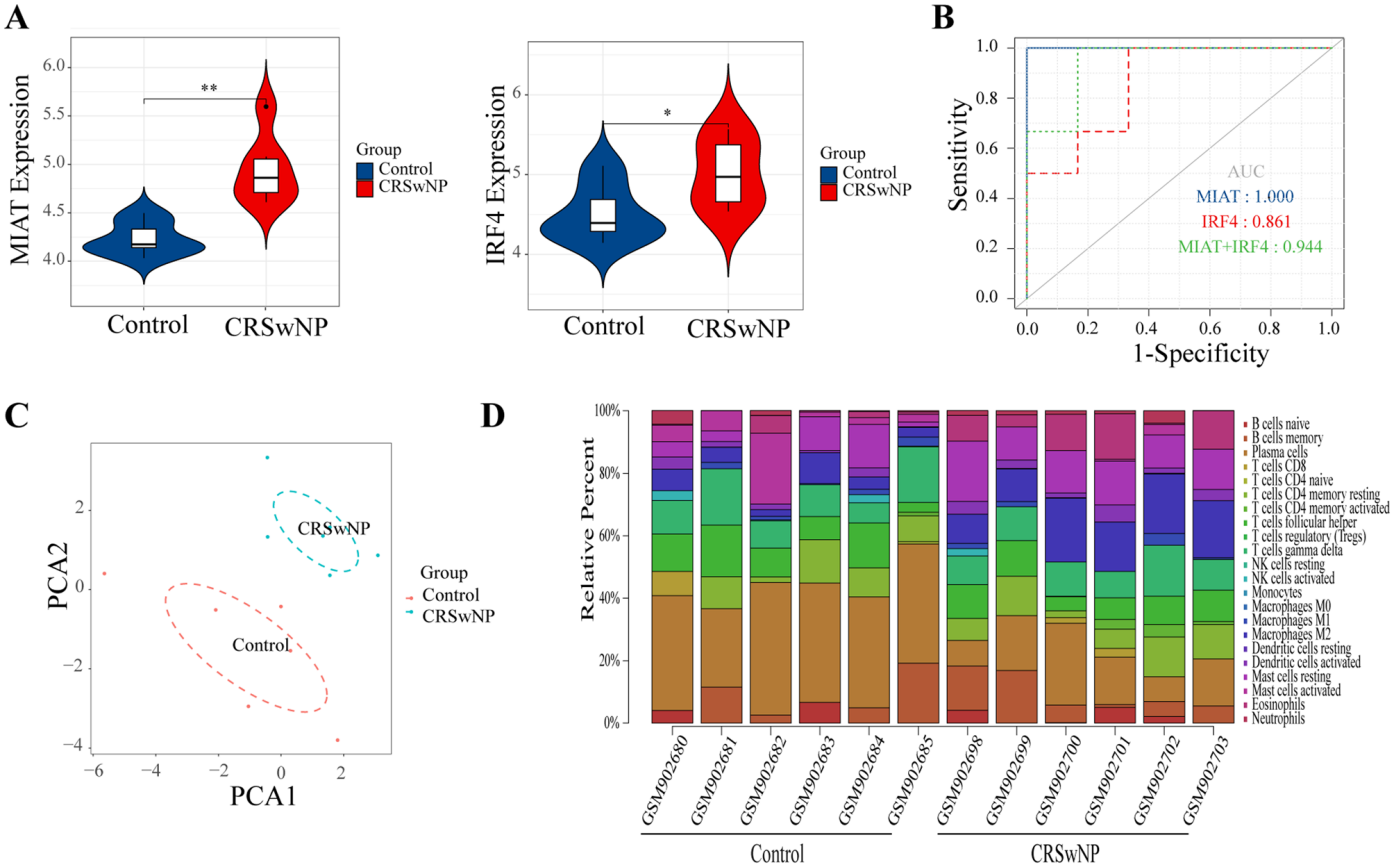
GSE36830 validation and evaluation of immune cell infiltration. (**A**) Expression pattern of MIAT and IRF4 in CRSwNP and control subjects. (**B**) The ROC curves of MIAT (blue dotted line), IRF4 (red dotted line), MIAT and IRF4 (green dotted line). (**C**) PCA cluster plot of immune cell infiltration performed on all samples. (**D**) Bar charts of 22 immune cell proportions in the CRSwNP and control groups. ROC, receiver operating characteristic; AUC, area under the receiver operating characteristic curve. * *p* value < 0.05, ** *p* value < 0.01.

To test the robustness of the lncRNA and mRNA biomarkers for CRSwNP, MIAT and IRF4 were applied to the independent GSE36830 dataset. As shown in Fig. 5A, the expression of MIAT (*p* value = 0.002) and IRF4 (*p* value = 0.041) was significantly upregulated in CRSwNP, which was consistent with our analyzed result. The diagnostic accuracy of MIAT and IRF4 was assessed using ROC curve analysis (AUC of MIAT: 1.000; AUC of IRF4: 0.861; AUC of MIAT and IRF4: 0.944) (Fig. 5B).

### Immune cell infiltration results

**Figure 6:**
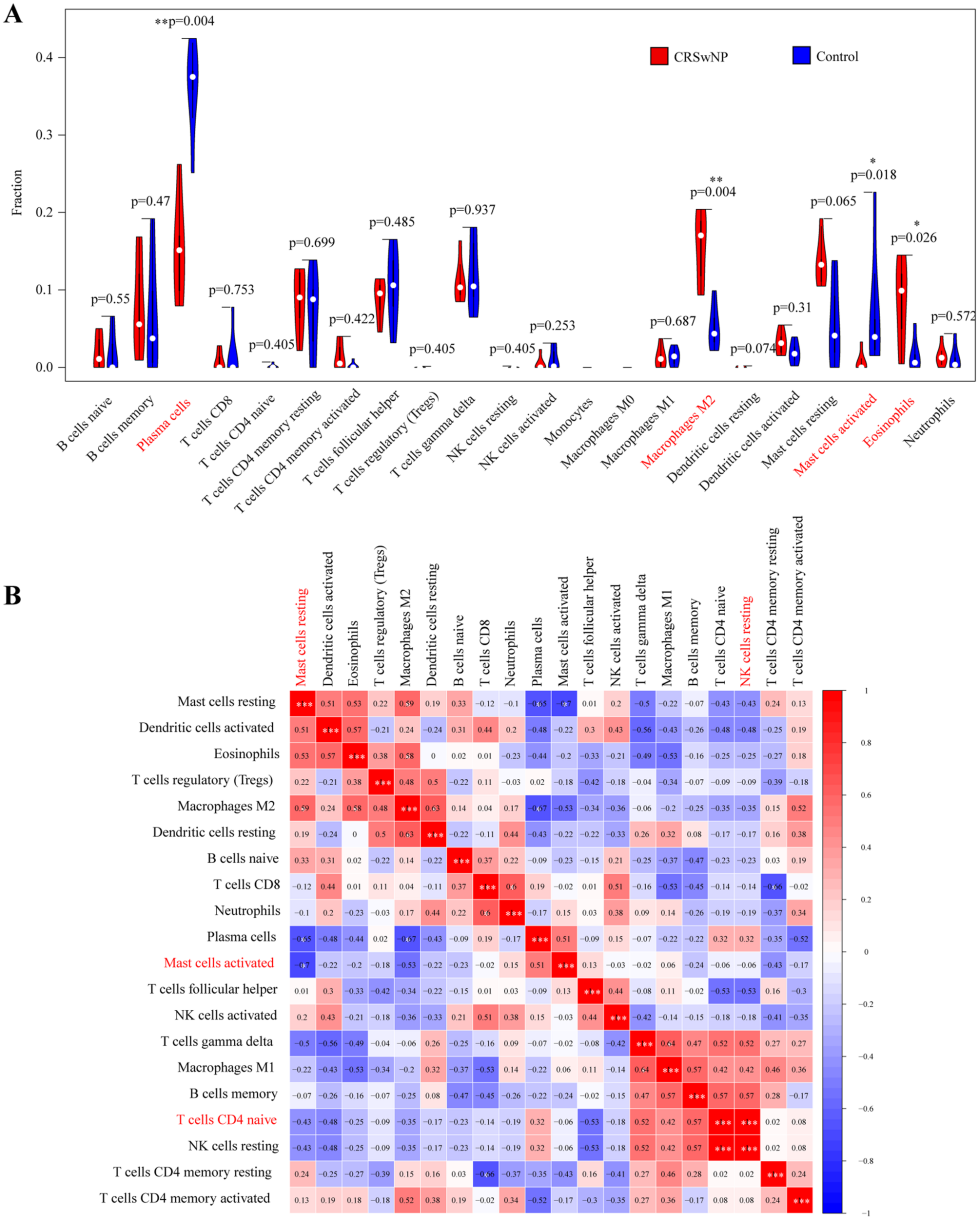
The landscape of immune infiltration between CRSwNP and normal controls. (**A**) Violin diagram of the proportion of 22 types of immune cells. (**B**) Correlation heatmap of 22 types of immune cells. Red: positive correlation; blue: negative correlation. Correlation heatmap were generated using R package “corrplot” (version 0.84. https://github.com/taiyun/corrplot). * *p* value < 0.05, ** *p* value < 0.01, *** *p* value < 0.001.

By using the CIBERSORT algorithm, we observed a difference in immune infiltration between CRSwNP tissues and normal tissues of 22 subpopulations of immune cells. Based on the criteria of *p* value < 0.05, 12 samples (6 CRSwNP samples and 6 control samples) were all included. As shown in Fig. 5C,D, the fraction of immune cells varied significantly among the CRSwNP and control samples. Then, the violin plot of the immune cell infiltration difference showed that eosinophils and M2 macrophages infiltrated more in CRSwNP samples than normal control samples. However, plasma cells and activated mast cells were relatively lower (Fig. 6A). Furthermore, a correlation heatmap of the 22 types of immune cells revealed that resting NK cells and naive CD4+T cells showed the most synergistic effect. Additionally, activated mast cells and resting mast cells showed the most competitive effect (Fig. 6B). Together, these results indicated that aberrant immune infiltration may have important clinical value in CRSwNP.

### Correlation analysis between the ceRNA axis (lncRNA and mRNA) and infiltrating immune cells

**Figure 7:**
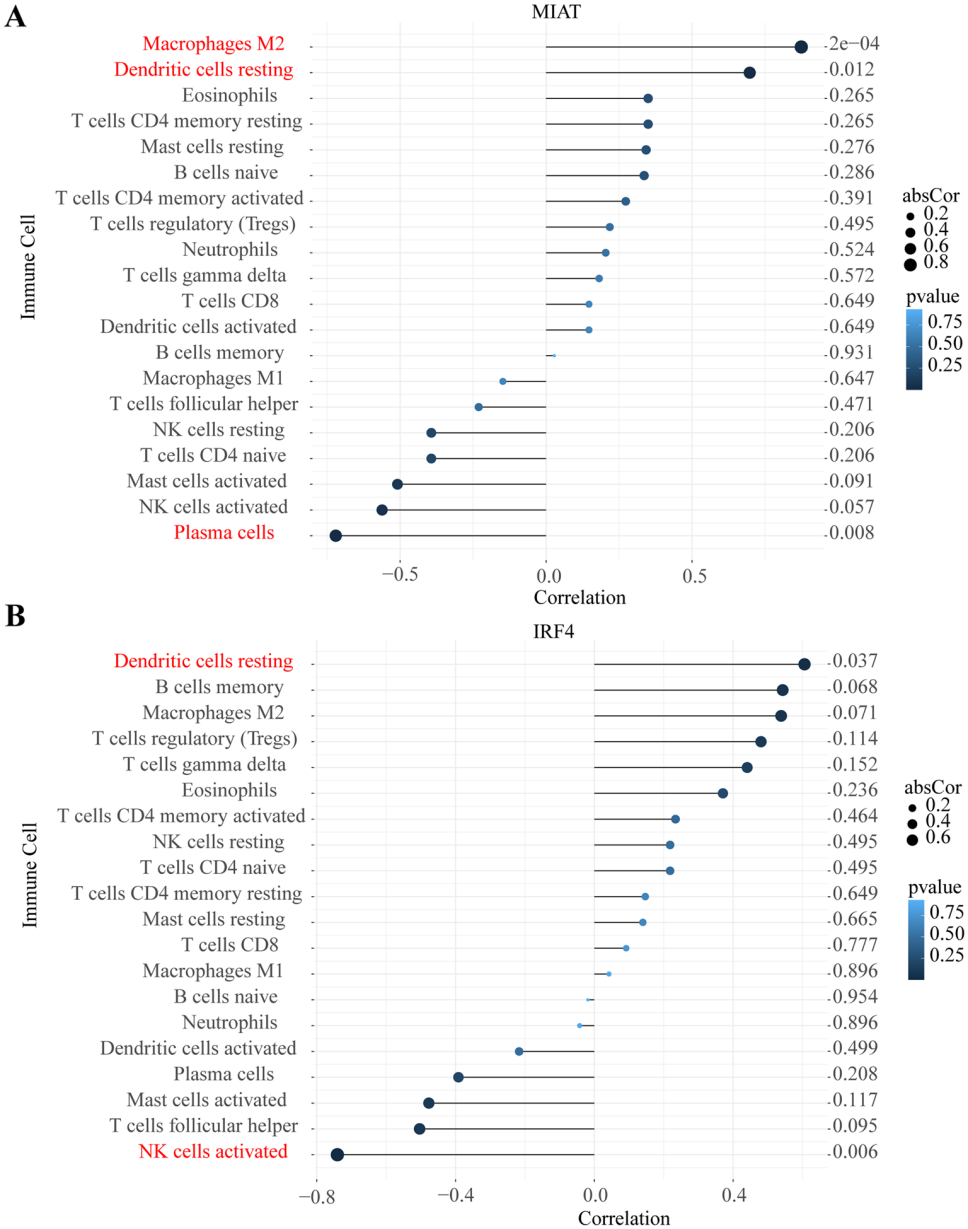
Correlation between the ceRNA axis (MIAT and IRF4) and infiltrating immune cells. (**A**) Correlation between MIAT and infiltrating immune cells. (**B**) Correlation between IRF4 and infiltrating immune cells.

Correlation analysis showed that MIAT was positively correlated with M2 macrophages (r = 0.874, *p* value < 0.001) and resting dendritic cells (r = 0.698, *p* value = 0.012) and negatively correlated with plasma cells (r = − 0.720, *p* value = 0.008) (Fig. 7A). IRF4 was positively correlated with resting dendritic cells (r = 0.606, *p* value = 0.037) and negatively correlated with activated NK cells (r = − 0.741, *p* value = 0.006) (Fig. 7 B). Therefore, these findings further support that the ceRNA axis is closely correlated with the immune infiltration level in CRSwNP, suggesting that the ceRNA regulatory network might be a novel target for the diagnosis and treatment of CRSwNP.

## Validation

### In-silico validation

**Figure 8:**
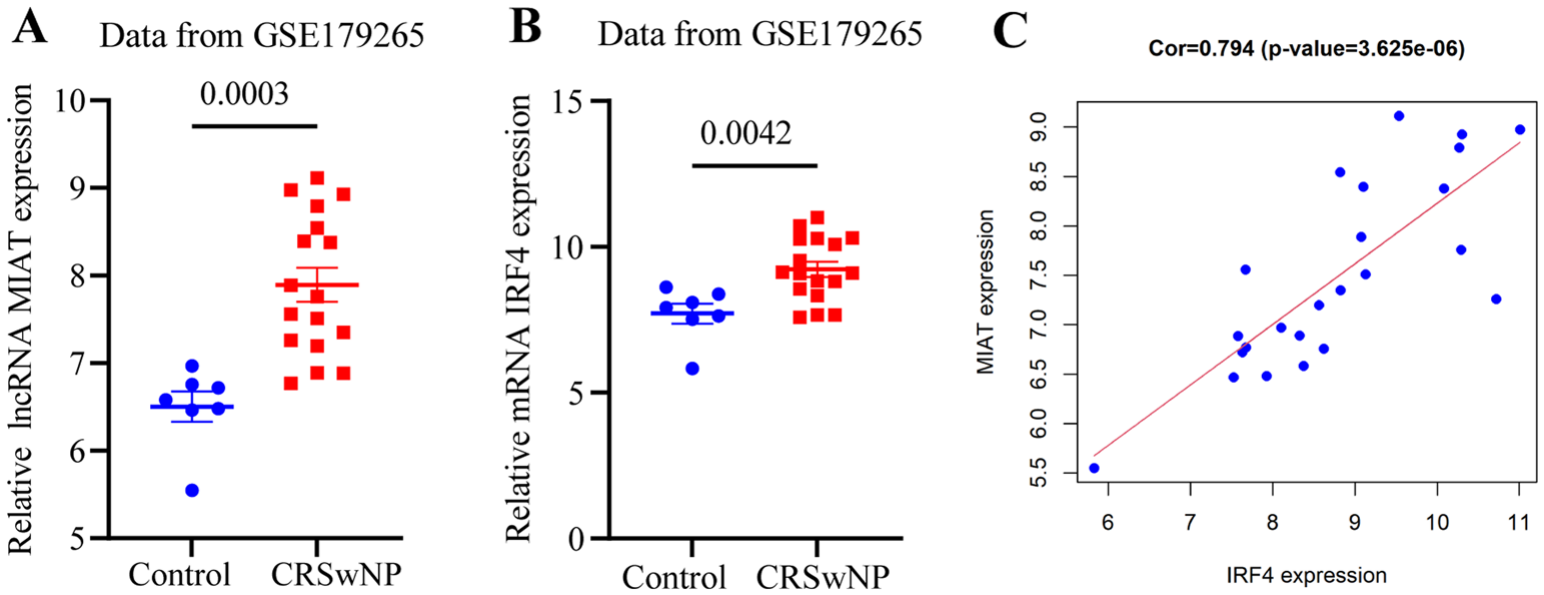
In-silico validation. (**A**) Expression pattern of MIAT in CRSwNP and control subjects. (**B**) Expression pattern of IRF4 in CRSwNP and control subjects. (**C**) Correlation analysis between MIAT and IRF4 in CRSwNP.

We performed in-silico validation by using another dataset. In GSE179265, the expression of MIAT (*p* value = 0.0003) and IRF4 (*p* value = 0.0042) significantly up-regulated in the nasal polyp group than control group (Fig. 8A,B). The expression of IRF4 was positive correlation with lncRNA MIAT (r = 0.794, *p* value < 0.05) (Fig. 8C).

### Experimental validation

**Figure 9:**
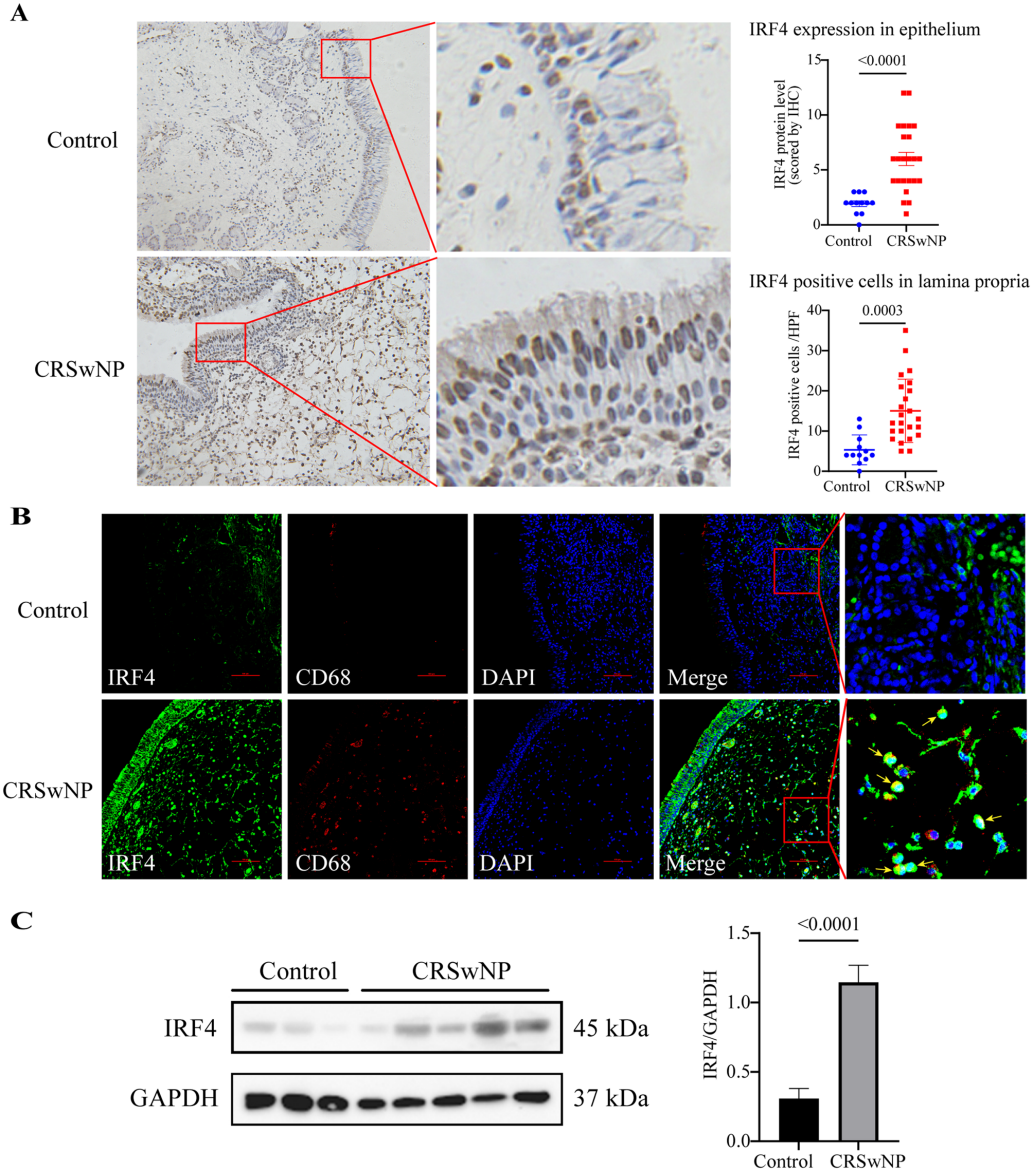
Experimental validation of the protein expression levels of IRF4. (**A**) Immunohistochemistry study demonstrated increased expression of IRF4 in the cytoplasm and nucleus, in nasal epithelial cells and inflammatory cells in lamina propria in nasal polyps. The representative photomicrographs are shown (original magnification × 200). (**B**) Double immunofluorescence staining demonstrated IRF4 expression in CD68 + macrophages in lamina propria in nasal polyps. Arrows denote representative positive cells. (**C**) Representative blots of IRF4 protein expression in sinonasal mucosa.

Immunohistochemistry showed that IRF4 is expressed in mucosal epithelium and macrophages of lamina propria in nasal polyp tissues, and the numbers of IRF4 positive cells in epithelium and lamina propria were marked enhanced in nasal polyp tissues in comparison with those in control tissues (Fig. 9A). Confocal showed co-localization of IRF4 and CD68 (M2 macrophage marker) in lamina propria (Fig. 9B). Western blot results showed that the expression of IRF4 in nasal polyps was significantly higher than that in the control group (*p* value < 0.0001) (Fig. 9C).

## Discussion

In the past, many studies have investigated the molecular mechanisms of nasal polyp formation and growth^22,49–51^. However, most studies have focused on elucidating the molecular mechanism involved in mRNAs and miRNAs^52,53^. With the rapid development of next-generation sequencing, long noncoding RNAs (lncRNAs) longer than 200 nucleotides in length have been discovered. This novel transcript has been demonstrated to play important roles in a wide range of biological processes in humans. Recent studies have demonstrated the vital role of lncRNAs in CRSwNP, and some of these lncRNAs may be potential biomarkers for diagnosis and therapeutic targets^32,54^. The competing endogenous RNA (ceRNA) hypothesis proposed by Salmena et al. in 2011 pointed to lncRNAs acting as endogenous molecular sponges that competitively bind miRNAs via shared MREs with reverse complementary binding seed regions to indirectly regulate mRNA expression levels^24^. Although a number of studies have indicated the involvement of ceRNA crosstalk in tumorigenesis^55^, progression^56^ and metastasis^57^ of cancers, there is still a paucity of studies on the comprehensive analysis of the lncRNA-miRNA-mRNA ceRNA network in CRSwNP.

CRSwNP is characterized by tissue remodeling (with collagen and fibrin deposition) and inflammation and has combinations of Th1, Th17 and/or Th2 inflammatory cytokines^58,59^. Dysregulation of the immune response, epithelial barrier dysfunction, microbial colonization, and environmental exposures have been reported as etiologic factors of CRSwNP^5,60^. The dysregulation of the host immune system has also been thought to play prominent roles in the pathogenesis of CRSwNP^15^. Defects in the innate immune function of the upper airway epithelium have been reported to play a role in the initial inflammatory response leading to CRSwNP. Subsequent recruitment and activation of eosinophils, neutrophils, mast cells, basophils, and innate lymphoid cells could further contribute to a chronic inflammatory response and directly activate adaptive immune cells, including T and B cells^61^. Therefore, finding specific diagnostic markers and analyzing the pattern of CRSwNP immune cell infiltration have profound significance for the treatment of CRSwNP patients.

In the present study, we integrated four GEO datasets for CRSwNP, which included mRNA, lncRNA, and microRNA expression. First, we identified 565 DE-lncRNAs (267 upregulated and 298 downregulated), 23 DE-miRNAs (1 upregulated and 22 downregulated), and 1799 DE-mRNAs (1071 upregulated and 728 downregulated) in CRSwNP samples from the GEO database (Fig. 2A–C). Then, by functional enrichment analysis, these DE-mRNAs were significantly enriched in leukocyte migration, extracellular structure organization and extracellular matrix organization (BP) (Fig. 3A). Notably, CCL18, CCL13, MMP11, SCG2, HAPLN1, and SPP1 were significantly upregulated genes in BP (Fig. 3B). CCL18 and CCL13 are chemokines; furthermore, CCL18 is regulated by Th2 cytokines and is significantly upregulated in CRSwNP^62^. Matrix metalloproteinase 11 (MMP11) belongs to the matrix metalloproteinase (MMP) family. MMP11 plays a role in different biological processes, such as embryonic tissue remodeling, extracellular matrix (ECM) remodeling, epithelial growth, and wound healing^63^. Secretogranin II (SCG2) has been shown to suppress endothelial cell apoptosis and enhance angiogenesis of endothelial cells to facilitate neo-vascularization and rapid wound repair and regeneration^64^. Hyaluronan and proteoglycan link protein 1 (HAPLN1) and secreted phosphoprotein 1 (SPP1) are associated with ECM structure, composition, and mechanics in cancer^65,66^. Subsequent KEGG-GSEA pathway enrichment analysis also suggested that those DE-mRNAs may be involved in various pathways related to CRSwNP, such as Staphylococcus aureus infection, osteoclast differentiation, and phagosomes (Fig. 3C). CRSwNP patients are significantly more likely to be colonized with Staphylococcus aureus than controls and proteins Staphylococcus aureus as inducers of persistent type 2 airway inflammation^67,68^. Neutrophils are essential for the clearance of bacteria either through phagocytosis or the formation of neutrophil extracellular traps (NETs)^69^. The associations of eosinophilic infiltrate with CRSwNP and higher osteitis scores have been demonstrated^70^. The inflammatory milieu disrupts the balance between osteoblasts and osteoclasts and leads to neo-osteogenesis^71^. Taken together, these results suggested that these DE-mRNAs are involved in CRSwNP biological processes and contribute to nasal polyps.

To better research the mechanism of CRSwNP, we first constructed a novel ceRNA regulatory network (including 21 lncRNAs, 8 miRNAs, and 8 mRNAs) involved in CRSwNP through bioinformatic analysis (Fig. 4B). Next, the hub lncRNA MIAT was obtained by hub analysis based on degree > 5 (Fig. 4C) (Table 2). Finally, based on coexpression of lncRNA-mRNA and its correlation analysis, a MIAT/miR-125a/IRF4 regulatory axis that fit the ceRNA pattern well was proposed (Fig. 4D,E). We verified the expression of MIAT and IRF4 in the extra validation GEO datasets (GSE36830 and GSE179265 ) (Figs. 5A,B and 8A,B), the upregulated expression of IRF4 was positive correlation with lncRNA MIAT (Fig. 8C), which further indicated that our screening results were reliable. MIAT was first identified in heart tissues and is known as myocardial infarction-associated transcript^72^. This lncRNA can regulate the expression of certain genes at the transcriptional or posttranscriptional levels. Constant studies have led to the fact that MIAT is involved in various diseases and cellular processes, mainly related to the activation of the inflammatory response^73^. One study showed that the expression of MIAT increased in LPS-induced pneumonia, and deletion of MIAT protected against LPS-induced inflammation via the miR-147a/NKAP/NF-κB axis^74^. Another study showed that the expression of MIAT increased in the knee joint synovium and heart muscles of arthritic mice and macrophage inflammation^75^. MiR-125a-5p has an important role in suppressing classical activation of macrophages while promoting alternative activation to promote anti-inflammatory responses^76^. MiR-125a-5p is involved in the molecular mechanisms of rheumatoid arthritis^77^ and osteoarthritis^78^. IRF4 is a transcription factor that is related to the regulation of gene expression and the immune response. IRF4 plays an essential role in T cells, B cells, and myeloid cell differentiation^79^. IRF4 expression is associated with many lymphoid malignancies and multiple myeloma^80^. Additionally, the expression of IRF4 is increased in rheumatoid arthritis patients compared with healthy volunteers^81^. GSEA of IRF4 identified several immune cell infiltration and immune response-related pathways (Fig. 4F).

IRF4, a major regulator of M2 macrophage activation, has been demonstrated to be involved in the pathogenesis of allergic rhinitis and athma^82,83^. In this study, we found that IRF4 was markedly expressed in epithelial cells as well as macrophages in the nasal polyp tissues (Fig. 9A,B). We found that the production of IRF4 was upregulated in nasal polyps (Fig. 9C). The results provide strong support for our computational analyses, which suggests that IRF4 may be a potential biomarker for the selection of therapeutic strategies for CRSwNP.

To further explore the role of immune cell infiltration in CRSwNP, the CIBERSORT website was used to perform a comprehensive evaluation of 22 types of immune cell infiltration in CRSwNP patients (Fig. 6A). We found that increased infiltration of eosinophils and M2 macrophages may be related to the occurrence and development of CRSwNP. CRSwNP in the Caucasian population was categorized by a type 2 inflammatory response with enhanced tissue eosinophilia and higher levels of type 2 cytokines (interleukin (IL)-4, IL-5, and IL-13)^15^. However, non-ECRSwNP is more common in Asian populations, which have lower levels of IL-5 and increased levels of the type 1 cytokine IFN-γ when compared to nasal polyps from European patients^84^. In the last two decades, there has been a trend toward increasing eosinophilic nasal polyps in Asian populations, possibly due to the rapid global adoption of a Western lifestyle^85^. ECRSwNP demonstrates Th2 inflammation with higher recurrence and asthma comorbidity rates and a more frequent and severe presentation of clinical symptoms than non-ECRSwNP (Th1/Th17 inflammation)^1^. Epithelial-derived innate cytokines, such as IL-25, IL-33, and thymic stromal lymphopoietin (TSLP), can stimulate eosinophils and their recruitment toward the impaired epithelium indirectly^86^. Interestingly, eosinophils are locally activated in nasal polyp tissue to secrete protein and inflammatory mediators contributing to tissue damage and remodeling and further eosinophil recruitment^87^. In addition to eosinophils, several other immune cell types are involved in the immunological mechanisms of CRSwNP, including macrophages, innate type 2 lymphoid cells, and mast cells^15^. Macrophages are phagocytic cells of the innate immune system, acting as a first line of defense along with the mucosal barrier. Activated macrophages are subdivided into proinflammatory (M1) and anti-inflammatory (M2) macrophages^88^. M1 macrophages support a Th1 response by expressing IL-1β, IL-6, IL-12, and TNF-α, while M2 macrophages contribute to tissue repair and promote Th2 responses^89^. There were positive correlations between the number of M2 macrophages and clinical disease severity and inflammation in ECRSwNP^90^. M2 macrophages can be alternatively activated by Th2 cytokines in tissue remodeling^91^. Our results further provided evidence that eosinophils and M2 macrophages could have possible therapeutic value in the treatment of patients with CRSwNP.

By analyzing the correlation between the ceRNA axis (lncRNA and mRNA) and immune cells, we found that MIAT was significantly positively correlated with resting dendritic cells (DCs) and M2 macrophages (Fig. 7A). IRF4 was significantly positively correlated with resting dendritic cells (Fig. 7B). MIAT acted as a ceRNA to regulate CD47 expression by sponging miR-149-5p in macrophages to inhibit efferocytosis in advanced atherosclerosis^92^. IRF4 is a critical transcription factor both for CD4+T cells and DCs^93,94^. IRF4 bound and activated IL-10 and IL-33 genes in DCs to promote Th2 differentiation and inflammation. DCs are used to bridge the innate and adaptive immune systems. DCs are innate immune cells that play a critical role in the host response to infection, as they recognize and respond to various antigens. Their defining role in adaptive immunity is to present exogenously introduced antigens in the context of MHC molecules for the activation of naive T cells^95^. In addition to antigen presentation, cytokine production by DCs could affect T-cell differentiation and function^94^. These results suggested that MIAT, IRF4, dendritic cells, and M2 macrophages play important roles in the immune response in patients with polyps. To the best of our knowledge, this is the first study to reveal the relationship between the ceRNA axis (lncRNA and mRNA) and infiltrating immune cells in CRSwNP. Our study revealed that the ceRNA network plays an important role in the pathogenesis of CRSwNP, which may represent potential diagnostic biomarkers and therapeutic targets. Our study also provided new insight into gene-immune cell regulatory mechanisms in CRSwNP. However, there are some limitations to this study. First, in this study, bioinformatics methods were used to identify differentially expressed genes (DEGs) between CRSwNP and normal tissues that can help reveal the pathological mechanism of CRSwNP at the genetic level, while Poliseno et al. observed that nondifferentially expressed RNAs may also contribute to the spongy mechanism^27^. We did not study these nondifferentially expressed ceRNAs in this study. Second, these datasets lacked clinical information, including comorbid asthma, IgE levels, Lund-Mackay CT scoring, and endoscopic nasal polyp scores, which could be used to assess the correlation between key genes and clinical features. Third, the number of samples used for the miRNA dataset was very low, and only lncRNA and mRNA biomarkers were validated in other datasets because there were no other publicly available miRNA datasets about the analyzed disease. Fourth, we did not analyze the relationship between miRNAs and ceRNAs (lncRNAs and mRNAs) due to the absence of the expression profiles on the same datasets for each RNA player. Finally, this study was based on open-access online databases and only experimentally validated the mRNA expression. In the future, we will validate the ceRNA network in cells and animal models.

## Conclusion

In this study, we successfully constructed a potential lncRNA-miRNA-mRNA regulatory network in CRSwNP from multiple dimensions to provide a comprehensive understanding of the detailed molecular mechanisms underlying the pathogenesis of CRSwNP. Importantly, we confirmed a potential regulatory axis in which MIAT could regulate the expression of IRF4 by sponging miR-125a in CRSwNP. We also found that eosinophils and M2 macrophages may be involved in the occurrence and progression of CRSwNP. MIAT and IRF4 was significantly positively correlated with dendritic cells and M2 macrophages. We suppose that the MIAT/miR-125a/IRF4 axis may exert a critical role in the development and progression of CRSwNP.
